# Integrated co-cultivation and subsequent esterification: Harnessing *Saccharomyces cerevisiae* and *Clostridium tyrobutyricum* for streamlined ester production

**DOI:** 10.1186/s13068-025-02698-3

**Published:** 2025-09-01

**Authors:** Katharina Oehlenschläger, Michaela Lorenz, Emily Schepp, Sarah Di Nonno, Dirk Holtmann, Roland Ulber

**Affiliations:** 1 Institute of Bioprocess Engineering, University of Kaiserslautern-Landau, Gottlieb-Daimler-Straße 49, 67663 Kaiserslautern, Germany; 2 Institute of Process Engineering in Life Sciences, Karlsruhe Institute of Technology, Kaiserstraße 12, 76131 Karlsruhe, Germany

**Keywords:** Ethyl butyrate, Ester, Co-culture, Enzymatic esterification, ISPR, *Clostridium tyrobutyricum*, *Saccharomyces cerevisiae*

## Abstract

The rising demand for natural products is accelerating research into sustainable methods for producing bio-based flavourings like ethyl butyrate. In this study, ethyl butyrate was successfully produced through the enzymatic esterification of butyric acid and ethanol, which were derived from the co-cultivation of *Clostridium tyrobutyricum* and *Saccharomyces cerevisiae*. Initial monoculture experiments with both strains were performed to investigate compromised fermentation conditions for co-cultivation. Based on these findings, anaerobic co-cultivation conditions were established at 37 °C and 150 rpm, with the pH controlled at 6. The effects of varying inoculation times in co-culture were examined, considering the solvent and acid tolerance of both strains. Due to the limited acid tolerance of *S. cerevisiae*, with significant inhibition at butyric acid concentrations above 10 g L¯^1^, a time-delayed inoculation with *C. tyrobutyricum* was implemented. In batch experiments, the final concentrations of butyric acid and ethanol were 13.98 ± 3.06 g L¯^1^ and 21.43 ± 1.66 g L¯^1^, respectively. Further enhancement of product concentrations was explored through a fed-batch cultivation strategy yielding up to 45.62 ± 3.82 g L¯^1^ of butyric acid and 18.61 ± 4.11 g L¯^1^ of ethanol. Ethyl butyrate was formed from the fermentation products by lipase-catalysed enzymatic esterification in a two-phase system through the addition of an organic phase. The ester concentration in the organic phase reached a maximum of 23.93 ± 0.68 g L¯^1^ (esterification yield 25%). This study presents a viable approach to the production of bio-based ethyl butyrate offering a sustainable alternative to traditional chemical synthesis methods.

**Graphical Abstract:**

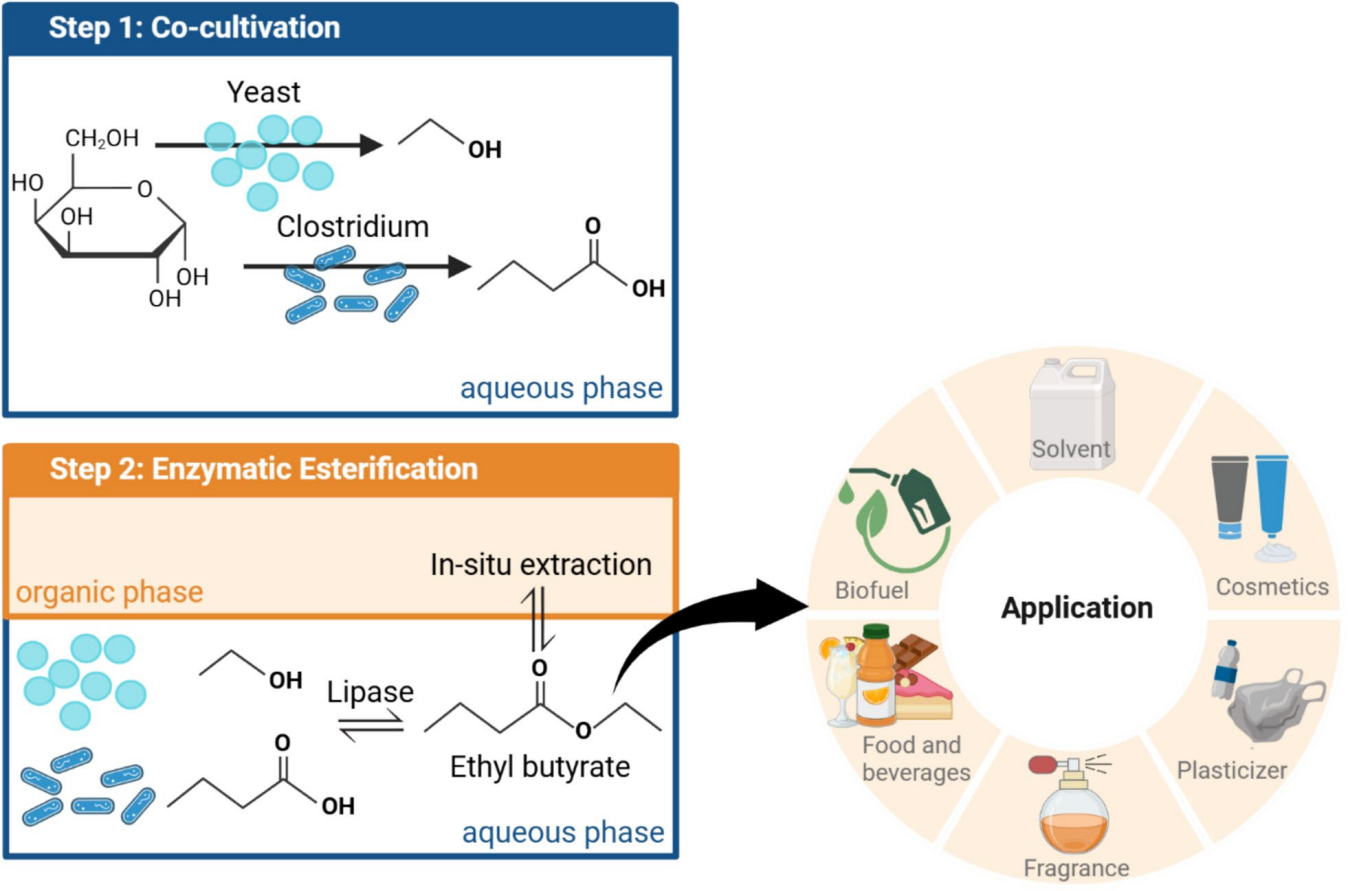

## Background

The fruity aromas associated with esters (e.g. isoamyl acetate, ethyl butyrate, and methyl butyrate) lead to their widespread use as flavouring agents in foods and beverages [56]. Beyond this application, esters are used as solvents, plasticizer and are considered promising candidates for biofuels. Fatty acid alkyl esters, derived from the transesterification of triglycerides from vegetable or animal sources, are already used as biodiesel [58]. Studies also emphasize the potential of ethyl butyrate and fermentation-derived ester blends as biofuel [9, 49].

Currently, low molecular weight esters are produced at multi-million-ton scales from fossil-based feedstocks [59]. To reduce greenhouse gas emissions and meet climate goals, it is essential to replace fossil resources with renewable raw materials and low-impact processes such as microbial or enzymatic synthesis. Since native microbial ester synthesis is limited to only a few microorganisms including *Saccharomyces cerevisiae*, *Kluyveromyces marxianus*, and *Yarrowia lipolytica*, which typically produce only low concentrations of esters, alternative microbial production strategies have emerged [8]. One such approach is the combination of microbial cultivation and enzymatic esterification of fermentation products [10, 33, 35, 48].

The combination of microbial cultivation with subsequent enzymatic esterification offers clear advantages over microbial de novo synthesis of esters, as both process steps can be specifically and independently optimized. The use of microbial co-cultures appears highly beneficial in the context of ester production, as it allows for the targeted synthesis of both carboxylic acid and alcohol substrates. Co-cultivation has already shown its potential in industrial bioprocesses, with applications in food production (e.g. cheese, yoghurt), wastewater treatment, and biofuel production [2, 13, 34]. The advantages of co-cultivation include extended product and substrate spectrum, increased product yield, and the reduction of inhibitory by-products [41, 46, 52, 60, 62]. Various combinations of Clostridium strains, such as cellulolytic with solventogenic or acetogenic with chain-elongating, have been successfully co-cultivated in several studies [3, 11, 28]. These co-cultivation approaches enable the efficient conversion of CO, CO₂, and syngas into ethanol and acetate, which can then be further processed into valuable medium-chain fatty acids [11]. The combination of oxygen-consuming yeasts like *Saccharomyces cerevisiae* with anaerobic organisms such as clostridia can further enhance process robustness. Here, *S. cerevisiae* depletes oxygen, creating anaerobic conditions favourable for clostridia [15, 36]. Co-culture approaches combining *Clostridium acetobutylicum* and *S. cerevisiae* take advantage of commensal and syntrophic interactions between the microorganisms to increase product titre and solvent tolerance and enable the use of a broader range of substrates [6, 34, 44].

Lipases are especially well-suited for esterification because of their high selectivity, ability to function under mild conditions, and broad substrate versatility, making them the most commonly used enzymes for ester synthesis [8, 51]. The enzyme lipase B from *Candida antarctica* (CALB) in immobilized form offers additional advantages, such as increased operational stability, simple recovery from the reaction medium, and allows for multiple cycles of enzyme reuse [22].

Building on these advantages, co-cultivating yeasts and clostridia presents a powerful strategy for enhancing bioprocess efficiency and product yield in processes in the production of biofuels and other valuable biochemicals. In this study, the co-cultivation of *S. cerevisiae* and *Clostridium tyrobutyricum* was established to investigate their metabolism and productivity in a co-cultivation. This system aims to produce ethanol and butyric acid, which are subsequently esterified to ethyl butyrate using immobilized lipase B from *Candida antarctica*, offering a promising route towards greener and more sustainable ester synthesis*.*

## Materials and methods

### Microorganisms

*Clostridium tyrobutyricum* DSM 2637 and *Saccharomyces cerevisiae* DSM 3799 were purchased from DSMZ (Deutsche Stammsammlung für Mikroorganismen und Zellkulturen, German Collection of Microorganisms and Cell Cultures GmbH, Braunschweig, Germany). Clostridia cells were stored in a 40% aqueous glycerol solution at -80 °C under anaerobic conditions. Cells of *S. cerevisiae* were stored aerobically under the same conditions.

### Cultivation conditions

Pre-cultures of *C. tyrobutyricum* were cultivated in serum flasks (200 mL) with a fermentation volume of 100 mL. As pre-culture medium, modified PY + X medium consisting of 5 g tryptone, 10 g yeast extract, 5 g peptone from meat, 5 g glucose, and 40 mL salt solution per litre was used. The salt solution contained 0.25 g CaCl_2_, 0.5 g MgSO_4_ × 7H_2_O, 1 g KH_2_PO_4_, 1 g K_2_HPO_4_, 10 g NaHCO_3_, and 2 g NaCl per litre [29]. The medium pH was adjusted to 5.5 by the addition of 1 M HCl solution. The medium was autoclaved and then flushed with nitrogen for 30 min to achieve anaerobic conditions. Inoculation was carried out by the addition of 1% v/v of cryo-culture. Pre-culture of clostridia was incubated at 37 °C and 50 rpm (Incubator Shaker Ecotron, Throw 25 mm, Infors HAT AG, Bottmingen, Switzerland) until late exponential growth phase was reached. Cell numbers of up to 4.58∙10⁸ ± 1.87∙10⁷ were determined in the pre-culture of *C. tyrobutyricum* by cell counting using a Neubauer counting chamber (Neubauer improved, Brand GmbH & Co. KG, Wertheim, Germany). *S. cerevisiae* was pre-cultured in 100 mL YPG medium in baffled shaking flasks (300 mL). YPG medium contained 10 g yeast extract, 20 g peptone and 20 g glucose per litre. Pre-cultures of yeast were incubated at 32 °C and 150 rpm for 17 h. Here, maximum cell numbers of 2.50∙10^8^ ± 8.63∙10^6^ were determined.

For main cultures MP2opt medium from Engel et al. was used, which is a medium optimized for clostridia cultivation [17]. This medium was used with an adapted nitrogen source. The adapted MP2optN medium consisted of 1.9 g (NH_4_)_2_SO_4_, 0.5 g KH_2_PO_4_, 0.5 g K_2_HPO_4_, 0.2 g MgSO_4_ × 7H_2_O, 0.015 g Fe(II)SO_4_ × 7H_2_O, 0.01 g MnSO_4_ × 7H_2_O, 1 mL of vitamin solution per litre. The vitamin solution contained 2 mg *p*-aminobenzoic acid, 2 mg thiamine–HCl and 0.01 mg D-biotin per litre. Glucose was added in a concentration of 50 or 100 g L^−1^. Product formation in mono- and co-culture experiments for both strains was analysed under anaerobic conditions. The medium was flushed with nitrogen prior to cultivation. Monocultures were inoculated by adding 10% v/v of respective pre-culture. Co-cultures of *C. tyrobutyricum* and *S. cerevisiae* were performed with the addition of 10% v/v of each strain. For the fed-batch experiments, 10 mL of a 500 g L⁻^1^ glucose solution was added at 24 and 48 h via syringe and cannula.

Experiments with pH regulation were conducted using a small-scale pH control system based on the setup described by Engel [16]. This setup consisted of 150-mL bottles (Duran, Wertheim, Germany) equipped with magnetic stirring bars and pH electrodes (SJ 114, VWR, Darmstadt, Germany). The pH regulation (± 0.1 pH accuracy) was carried out via an aquaristic control unit (ProfiLux 4, GHL, Kaiserslautern, Germany). The pH was controlled by the addition of 1 M NaOH. Optical density (OD), substrate and product concentrations were measured periodically by taking samples through a septum with a syringe and cannula. After quantification of the OD, the samples were centrifuged (centrifuge 5418, rotor FA-45-18-11, 16873 g, Eppendorf SE, Hamburg, Germany) for 2 min and sterile-filtered (Chromafil Xtra PA20/13, Macherey Nagel, Düren, Germany). Samples were stored at − 20 °C for further analysis.

The growth of *C. tyrobutyricum* at different ethanol concentrations was investigated in 200-mL serum flasks placed in a shaking incubator, equipped with a cell growth quantifier (Aquila Biolabs, Baesweiler, Germany) that measures the backscatter intensity. Growth rates were calculated with the DOTS Software (Aquila Biolabs, Baesweiler, Germany). Because of the limited capacity of slots in the cell growth quantifier, this measurement was performed without replicates. The product formation under ethanol stress conditions was studied in a separate shaking incubator in serum flasks in replicates. The influence of pH on butyric acid tolerance of *S. cerevisiae* was also measured in a cell growth quantifier. To facilitate yeast growth, this experiment was performed in shaking flasks.

### Esterification experiments

Untreated fermentation broth of the fed-batch experiment in co-culture was used for esterification experiments. Esterification was performed in 50-mL falcons using 20 mL fermentation broth. The pH of the fermentation broth was adjusted to 4 and 6 with 2 M HCl. Hexadecane was added in a volumetric ratio of 2:1 (aqueous phase:organic phase). Lipase B from *Candida antarctica* (Novozyme 435, lipase acrylic resin, ≥ 5000 U g^–1^, recombinant, expressed in *Aspergillus niger,* Merck KGaA, Darmstadt, Germany) was added in a concentration of 0.5 g L^–1^ relative to the aqueous phase to initiate the esterification reaction. Esterification experiments were performed in an incubation shaker at 150 rpm and 37 °C. Both phases were sampled periodically and stored at − 20 °C. Partitioning coefficients were determined at 37 °C based on the method of Zhang et al. [61].

### Analytical methods

Aqueous samples were analysed by high-performance liquid chromatography (HPLC) measurement. The HPLC system consisted of a Merck Hitachi L-6200 pump (Merck KGaA, Darmstadt, Germany), a Midas cool autosampler (Spark Holland B.V. Emmen, The Netherlands), a Jetstream II plus column thermostat (Duratec, Hockenheim, Germany), an Aminex HPX-87H column (300 mm × 7.8 mm, Bio-Rad Laboratories GmbH, Feldkirchen, Germany), and a RI detector (PN3140, Postnova, Landsberg am Lech, Germany). The mobile phase used was 2.5 mM H_2_SO_4_ at a flow rate of 0.6 mL min^−1^. The column temperature was set to 80 °C. To determine the OD, samples were analysed with the photometer Lambda Bio + (Perkin Elmer, Rodgau, Germany). Yields were calculated based on the amount of glucose consumed. For the co-culture, yields were based on the sum of ethanol and butyric acid concentrations. For the analysis of organic samples, a gas chromatography (GC) measurement was performed. Here, a PerkinElmer Clarus 500 Gas Chromatograph with flame ionization detector (FID) (Perkin Elmer, Rodgau, Germany) was used with a Stabilwax DA 4 column (capillary column, 15 m, 0.32 mm ID, 0.25 μm (Restek GmbH, Bad Homburg, Germany). Helium was used as carrier gas with a flow rate of 2 mL min^−1^. The oven temperature was increased from 40 °C to 225 °C at a rate of 30 °C per minute. Three biological replicates were used to calculate the standard deviation.

## Results

In order to establish a co-culture of *C. tyrobutyricum* and *S. cerevisiae,* preliminary experiments were carried out with monocultures of the two strains. Medium and cultivation conditions were studied in monocultures to determine conditions for co-cultivation. Also, the tolerance of each strain to the fermentation products butyric acid and ethanol was investigated. Based on these results, the co-culture was studied using different inoculation variants. The fermentation products ethanol and butyric acid were subsequently enzymatically esterified in the unprocessed fermentation broth to form ethyl butyrate.

### Monoculture of *Clostridium tyrobutyricum*

The production of butyric acid by *C. tyrobutyricum* has been the focus of numerous studies [18, 31, 38, 40, 50, 55]. The ideal cultivation conditions are usually maintained at pH 6, 37 °C, and 150 rpm with anaerobic conditions. According to this, a pH of 6 is favoured for butyric acid production with *C. tyrobutyricum*, whereas *S. cerevisiae* prefers lower pH around 4 [5, 63]. To assess the pH tolerance of *C. tyrobutyricum*, the pH was reduced to 5.5 and 5 (Table 1).

**Table 1:** Impact of pH on growth and production of *C. tyrobutyricum*

pH	max. cell density (OD_600_)	Butyric acid/g L¯^1^	Acetic acid/g L¯^1^	Yield_P/S_/g_BA_ g_Glu_¯^1^	Glucose consumption/%
6	18.17 ± 2.24	30.04 ± 4.00	6.06 ± 0.36	0.38 ± 0.03	87.28 ± 0.09
5.5	5.38 ± 3.01	8.36 ± 3.50	1.24 ± 0.48	0.28 ± 0.05	23.87 ± 2.56
5	3.32 ± 0.45	2.80 ± 0.55	1.27 ± 0.16	0.20 ± 0.08	14.94 ± 3.52

BA: butyric acid; Glu: glucose

Cultivation conditions: 150 rpm, 37 °C, V = 100 mL, controlled pH, inoculum size: 10 vol. %, c_0,glucose_ = 100 g L^−1^, n_biol._ = 3

Controlling the pH to 6 results in a butyric acid concentration of 30.04 ± 4.00 g L^–1^ and a yield of 0.38 ± 0.03 g g^–1^. These results are comparable with literature data using the same cultivation conditions and a glucose concentration of 100 g L^–1^ [38, 50]. A reduction in pH leads to a decrease in biomass and butyric acid production. At pH 5, butyric acid concentration decreases by 90.69 ± 2.22%, while OD is reduced by 81.73 ± 3.35%. Thus, a deviation from the optimum pH results in a significant reduction in butyric acid production by *C. tyrobutyricum*.

**Fig. 1:**
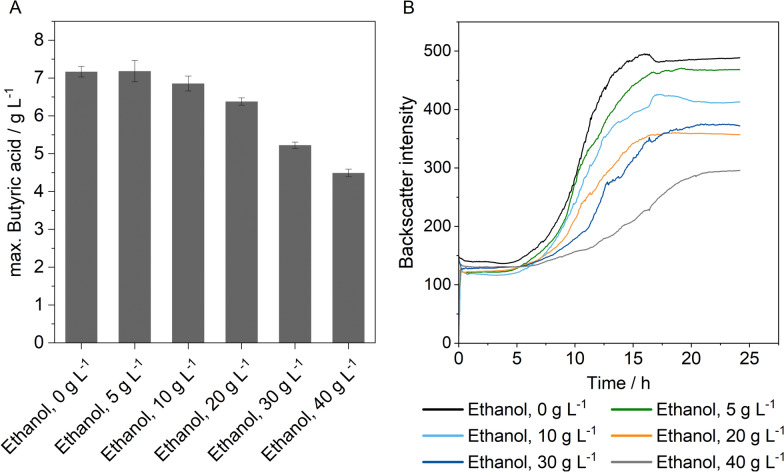
Ethanol tolerance of *C. tyrobutyricum*. **A** Effect on butyric acid production. Cultivation conditions: 150 rpm, 37 °C, V = 100 mL, inoculum size: 10 vol.%, pH_0_ = 6 (uncontrolled), *c*_0,glucose_ = 50 g L^*–*1^, n_biol._ = 3. **B** Effect on growth. Cultivation conditions: 150 rpm, 37 °C, V = 50 mL, inoculum size: 10 vol.%, pH_0_ = 6 (uncontrolled), n_biol._ = 1

As *C. tyrobutyricum* will be cultivated in co-culture with the ethanol producer *S. cerevisiae,* it is important to assess the ethanol tolerance of *C. tyrobutyricum* (Fig. 1)*.* For this, different concentrations of ethanol were supplemented to the cultivation medium. Measurements in a cell growth quantifier were performed to examine the impact of ethanol on the growth of *C. tyrobutyricum* (Fig. 1B).

A maximum butyric acid concentration of 7.17 ± 0.14 g L^–1^ was produced in the control experiment with no addition of ethanol (Fig. 1A). This relatively low concentration can be explained by the absence of a pH control within the experimental setup but still serves as a reference value for butyric acid production. An increase in the ethanol concentration leads to a reduction in the final butyric acid concentration. However, at an ethanol concentration of 40 g L^–1^, more than 60% butyric acid can still be found in comparison to the control cultivation. The same result can be seen for growth of *C. tyrobutyricum* (Fig. 1B)*.* Increasing the ethanol concentration leads to a decrease in growth rate and a reduction of the final cell density, indicated by a lower backscatter intensity. The maximum growth rate decreased by 62.46%, from 0.29 h^–1^ to 0.11 h^–1^, upon addition of 40 g L^–1^ ethanol.

### Monoculture of *Saccharomyces cerevisiae*

**Fig. 2:**
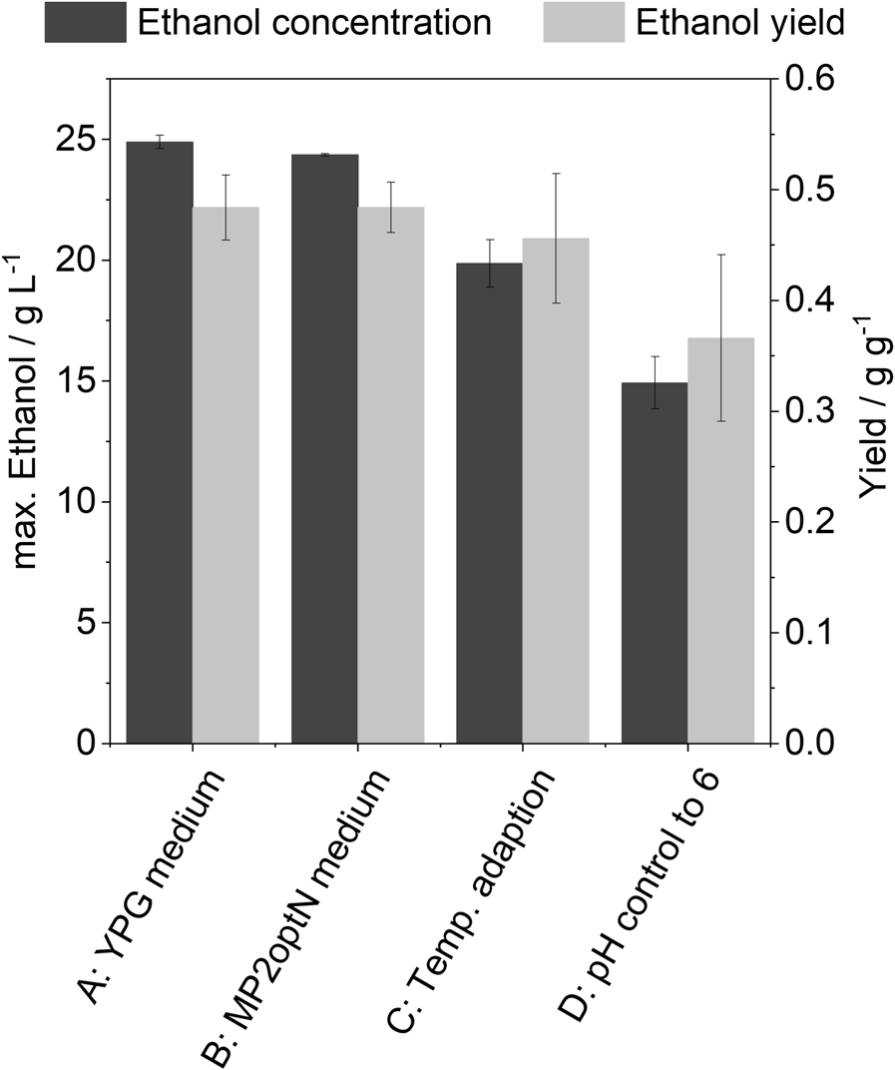
Cultivation of *S. cerevisiae* under adapted conditions. Cultivation conditions: 50 g L^*–*1^ glucose, V = 100 mL. **A** Cultivation in YPG medium, 32 °C, 150 rpm; **B** cultivation in MP2optN medium, 32 °C, 150 rpm; **C** cultivation in MP2optN medium, 37 °C, 150 rpm; **D** cultivation in MP2optN medium, 37 °C, 150 rpm, pH control to 6

To obtain combined cultivation conditions for co-culture, cultivation conditions for *C. tyrobutyricum* were transferred to the cultivation of *S. cerevisiae*. This includes the use of the clostridia medium MP2optN as well as the temperature and pH control required for butyric acid production. To evaluate the effect of the deviating cultivation conditions on ethanol production with *S. cerevisiae*, a comparison to the optimal cultivation conditions was conducted (Fig. 2). Optimal cultivation conditions for *S. cerevisiae* were determined according to the literature as 32 °C, 150 rpm, and no control of the pH [45].

Initially, cultivation in the adapted medium was considered. For this purpose, the ethanol production in the complex YPG medium (Fig. 2A) was compared to the production in MP2optN medium (Fig. 2B), which is a defined medium optimized for the cultivation of clostridia. Ethanol concentrations of 24.90 $\pm$ 0.27 g L^–1^ and 24.36 $\pm$ 0.04 g L^–1^, respectively, were measured in the YPG and in the MP2optN medium under anaerobic conditions, resulting in an ethanol yield of 0.48 g g^–1^. Based on these results, the MP2optN medium was classified as suitable for the cultivation of *S. cerevisiae,* as well as for co-cultivation. In a next step the cultivation conditions of the clostridia fermentation were transferred to the cultivation of *S. cerevisiae.* These include the adaption of the temperature to 37 °C (Fig. 2C) and the control of the pH to 6 (Fig. 2D). Both adaptions lead to a reduction of ethanol concentration and yield. Exceeding the optimum temperature results in a decrease in ethanol concentration and yield of 18.43 ± 4.02% and 4.95 ± 12.91%, respectively. The lowest ethanol concentration was measured when pH was additionally controlled to 6, resulting in a final ethanol concentration of 14.93 ± 1.40 g L^–1^. The ethanol yield decreased to 0.37 ± 0.07 g g¯^1^.

**Fig. 3:**
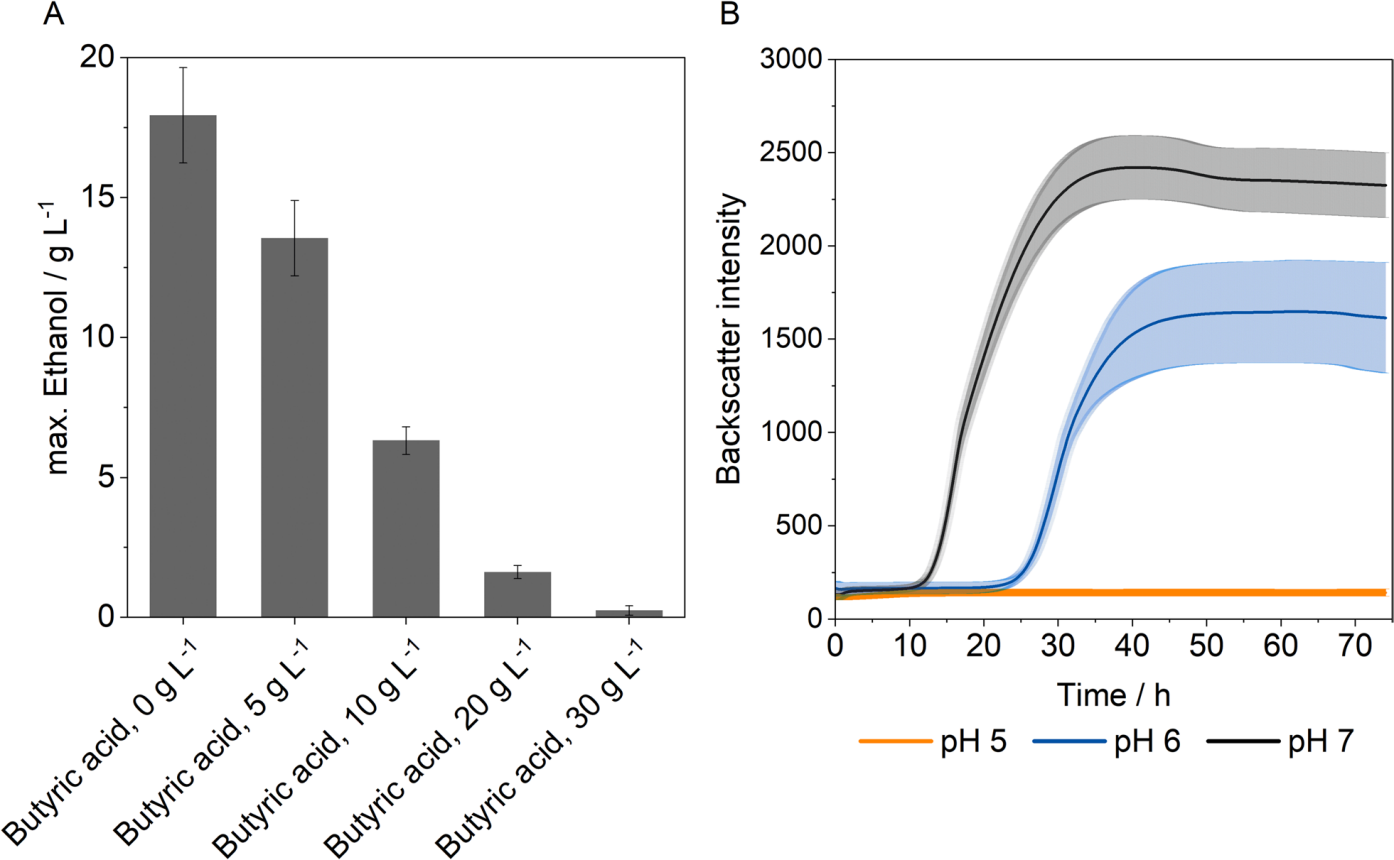
Butyric acid tolerance and pH tolerance of *S. cerevisiae*. **A** Effect of butyric acid concentration on ethanol production, cultivation conditions: 150 rpm, 37 °C, V = 100 mL, anaerobic conditions, inoculum size: 10 vol.%, pH_0_ = 6, c_0,glucose_ = 50 g L^*–*1^, n_biol._ = 3. **B** Effect of pH on growth. Cultivation conditions: 150 rpm, 37 °C, V = 50 mL, aerobic conditions, inoculum size: 10 vol.%, addition of butyric acid 5 g L^*–*1^, n_biol._ = 3

Butyric acid tolerance of *S. cerevisiae* is an important factor for the co-cultivation with clostridia. In order to study this aspect, the fermentation medium was supplemented with different concentrations of butyric acid. The effect on ethanol production was investigated under anaerobic conditions (Fig. 3A). The pH was adjusted to 6 at the beginning of the fermentation.

When butyric acid was added in a concentration of 10 g L^–1^, the final ethanol concentration was lowered by around 60%. Increasing the butyric acid concentration to 30 g L^–1^ leads to almost complete inhibition of ethanol production. To investigate the connection between pH and the inhibitory effect of the acid, the influence of 5 g L^–1^ butyric acid on growth of *S. cerevisiae* was evaluated at different pH levels in shaking flasks (Fig. 3B). Complete inhibition of growth occurs at a pH of 5. However, at a pH of 6, cell growth is observed, but a lag phase of 24 h is noticeable. A higher pH reduces the lag phase and results in an increased final cell density, highlighting a clear link between pH and butyric acid toxicity.

### Development of a co-cultivation method for *C. tyrobutyricum *and* S. cerevisiae*

Taking into account the preliminary experiments, a method for the co-cultivation of *C. tyrobutyricum* and *S. cerevisiae* was developed. The cultivation conditions were defined as pH of 6, 37 °C, and 150 rpm. The inoculation was performed using 10 vol.% of each strain.

As both strains show different tolerances to fermentation products, it might be useful to inoculate at different times. To investigate this, the times of inoculation with clostridia and yeast were varied according to Table 2.

**Table 2:** Variation of inoculation time for co-cultivation of *S. cerevisiae* and *C. tyrobutyricum* compared to results in monoculture

Inoculation variant	Ethanol/g L^¯1^	Butyric acid/g L^¯1^	Yield_P/S_*/g_BA+EtOH_ g_Glu_^¯1^	Glycerol/g L^¯1^	Acetic acid/g L^¯1^	Glucose consumption/%
Monoculture *S. cerevisiae* (Fig. 2D)	14.93 ± 1.40	–	0.31 ± 0.05	–	0.68 ± 0.12	85.73 ± 9.99
Monoculture *C. tyrobutyricum*	–	14.07 ± 2.55		–	3.87 ± 0.24	99.91 ± 0.13
*S. cerevisiae* at 0 h *C. tyrobutyricum* at 0 h	10.62 ± 0.73	22.38 ± 0.42	0.35 ± 0.01	5.86 ± 0.32	1.52 ± 1.30	92.66 ± 2.84
*S. cerevisiae* at 0 h *C. tyrobutyricum* at 24 h	21.43 ± 1.66	13.98 ± 3.06	0.29 ± 0.02	12.21 ± 3.74	1.27 ± 0.98	99.74 ± 0.25
*S. cerevisiae* at 24 h *C. tyrobutyricum* at 0 h	0.58 ± 0.06	19.45 ± 0.51	0.24 ± 0.03	0.09 ± 0.03	3.93 ± 1.07	81.04 ± 1.47

Cultivation conditions: 150 rpm, 37 °C, V = 100 mL, anaerobic conditions, inoculum size: 10 vol.% each strain, pH = 6, c_0,glucose,co-culture_ $\approx$ 100 g L^−1^, c_0,glucose,monoculture_ $\approx$ 50 g L^−1^, n_biol._ = 3

^*^Yield: the combined yield of ethanol and butyric acid is calculated based on the total glucose consumption

**Fig. 4:**
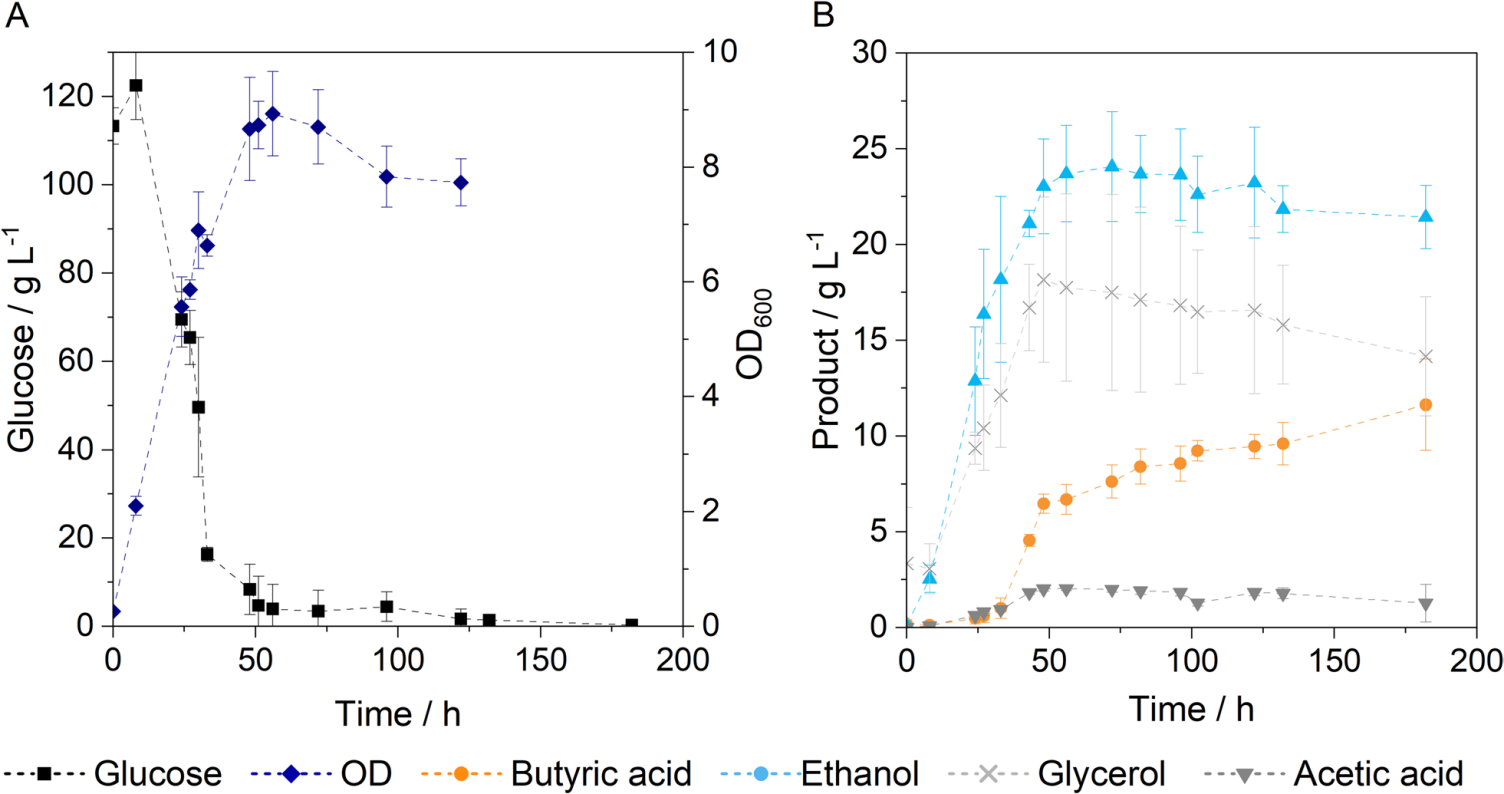
Co-cultivation of *S. cerevisiae* and *C. tyrobutyricum*. Cultivation conditions: 150 rpm, 37 °C, V = 100 mL, anaerobic conditions, inoculum size each: 10 vol.%, inoculation with *S. cerevisiae* at 0 h, inoculation with *C. tyrobutyricum* at 24 h, pH = 6, n_biol._ = 3

The simultaneous inoculation of both strains yields approximately twice the concentration of butyric acid compared to ethanol. This resulted in the highest yield of 0.35 ± 0.01 g g^–1^, calculated as the sum of ethanol and butyric acid production relative to the glucose consumed. This yield is comparable to the combined product yield obtained in the respective monocultures. However, the simultaneous formation of both products leads to an early inhibition of the yeast and limits the ethanol production, a finding that aligns with the expectations set by preliminary experiments. Consequently, the inoculation of *C. tyrobutyricum*, followed by *S. cerevisiae* after 24 h, completely suppresses ethanol production. To enhance ethanol production, the inoculation with *C. tyrobutyricum* can be delayed by 24 h. Starting with the inoculation of *S. cerevisiae* results in a higher ethanol concentration of 21.43 ± 1.66 g L^–1^ and a butyric acid concentration of 13.98 ± 3.06 g L^–1^. For better understanding of dynamics in co-culture, the course of fermentation performed by time-delayed inoculation with *C. tyrobutyricum* is shown in Fig. 4.

**Fig. 5:**
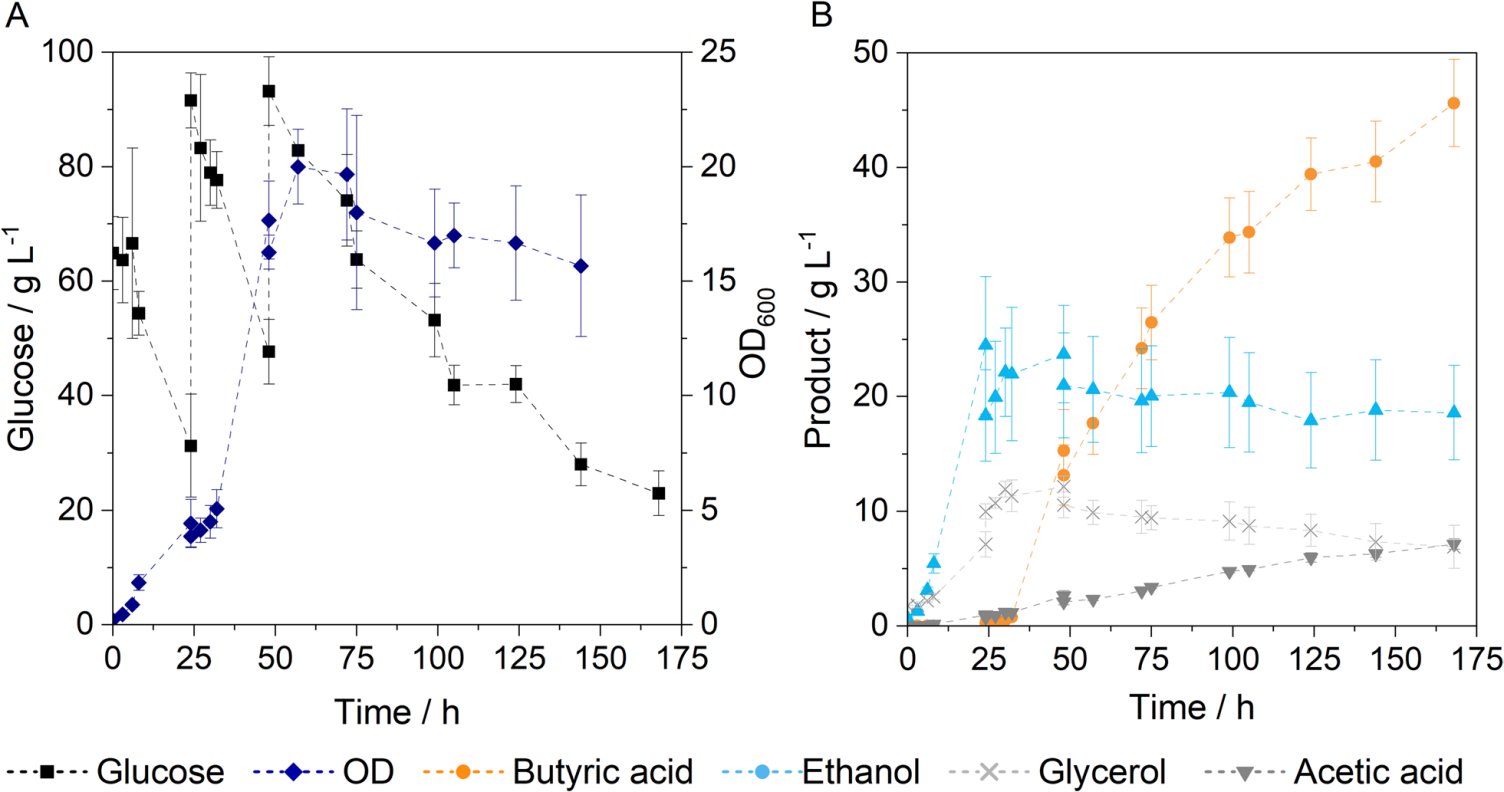
Fed-batch co-cultivation of *S. cerevisiae* and *C. tyrobutyricum*. Cultivation conditions: 150 rpm, 37 °C, V = 100 mL, anaerobic conditions, inoculum size each: 10 vol.%, inoculation with *S. cerevisiae* at 0 h, inoculation with *C. tyrobutyricum* after 24 h, feeding at 24 and 48 h, pH = 6, n_biol._ = 3

Cultivation starts with the inoculation of *S. cerevisiae* resulting in fast ethanol production and a maximum ethanol concentration of 24.06 g L^–1^ that is reached after 72 h. After 24 h, *C. tyrobutyricum* is added, by which time about half the sugar has been consumed. No inhibition of ethanol production is observed, as ethanol continues to be produced until the sugar is nearly fully consumed. Butyric acid production from clostridia starts without any lag phase and reaches a final concentration of 13.98 ± 3.06 g L^–1^. Despite the complete breakdown of the sugar after about 72 h, butyric acid formation can still be observed. The formation of butyric acid after 72 h is associated with the degradation of glycerol. The optical density increases continuously until the sugar is depleted and reaches a maximum of 8.93 ± 0.65. Throughout the cultivations, glycerol and acetic acid were detected as by-products, with glycerol found in particularly high concentrations of up to 18 g L^–1^. A fed-batch strategy may help reduce glycerol formation while enabling higher butyric acid production. For this reason, a fed-batch was carried out, whereby glucose (c $\approx$ 50 g L^−1^) was added after 24 and 48 h of fermentation (Fig. 5).

A maximum ethanol concentration of 24.49 ± 6.00 g L^−1^ was measured after 24 h. A decrease in ethanol concentration due to dilution effects is observed with the further addition of glucose. Ethanol production ceases after 48 h, coinciding with a butyric acid concentration of approximately 15 g L^–1^. The maximum glycerol concentration could be reduced to 12.17 ± 0.91 g L^–1^ by adjusting the feed strategy, but still high glucose concentrations are reached, so further optimization is necessary. However, with the fed-batch strategy, a significant increase in butyric acid concentration was observed, resulting in a final acid concentration of 45.62 ± 3.82 g L^–1^. From the sum of the main products, a mixed yield of 0.50 ± 0.04 g g¯^1^ can be calculated, which is higher in comparison to the batch experiment.

### Esterification of ethanol and butyric acid

**Fig. 6:**
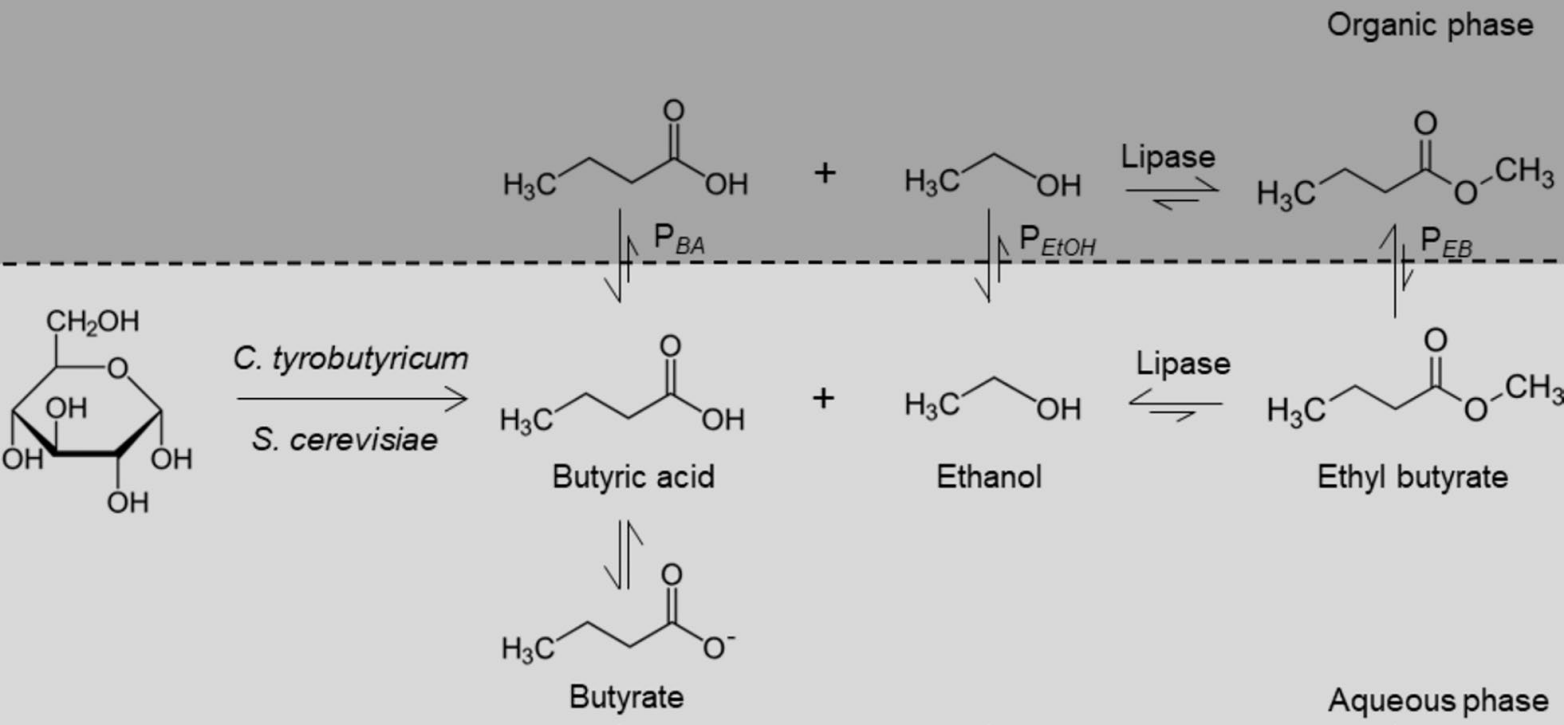
Schematic representation of the distribution and reaction equilibrium for the enzyme-catalysed esterification of butyric acid and ethanol to produce ethyl butyrate in a two-phase system. P: partition coefficient of the respective substance; BA: butyric acid; EB: ethyl butyrate; EtOH: ethanol. The length of the arrow indicates the relative tendency of the reaction and the distribution. Modified based on van den [53]

Building on the fermentation results, the unprocessed fermentation broth containing ethanol and butyric acid at concentrations of 18.61 ± 4.10 g L^–1^ and 45.62 ± 3.82 g L^–1^, respectively, was used as a substrate for esterification to produce the higher-value ester ethyl butyrate. Lipase B from *Candida antarctica* is a popular lipase for esterification due to its high catalytic activity in different environments. Its strong positional and stereoselectivity reduces by-product formation, enhancing the purity and yield of the desired ester, as highlighted in a recent review by Wang et al. [54]. Due to the competing hydrolysis reaction, enzyme-catalysed ester synthesis is unfavourable in aqueous solutions [37]. It is therefore necessary to add an organic phase to facilitate the formation of the ester. In the two-phase system, the esterification reaction is influenced by the extraction equilibrium, which is determined by the distribution coefficients of the reactants (Fig. 6).

**Fig. 7:**
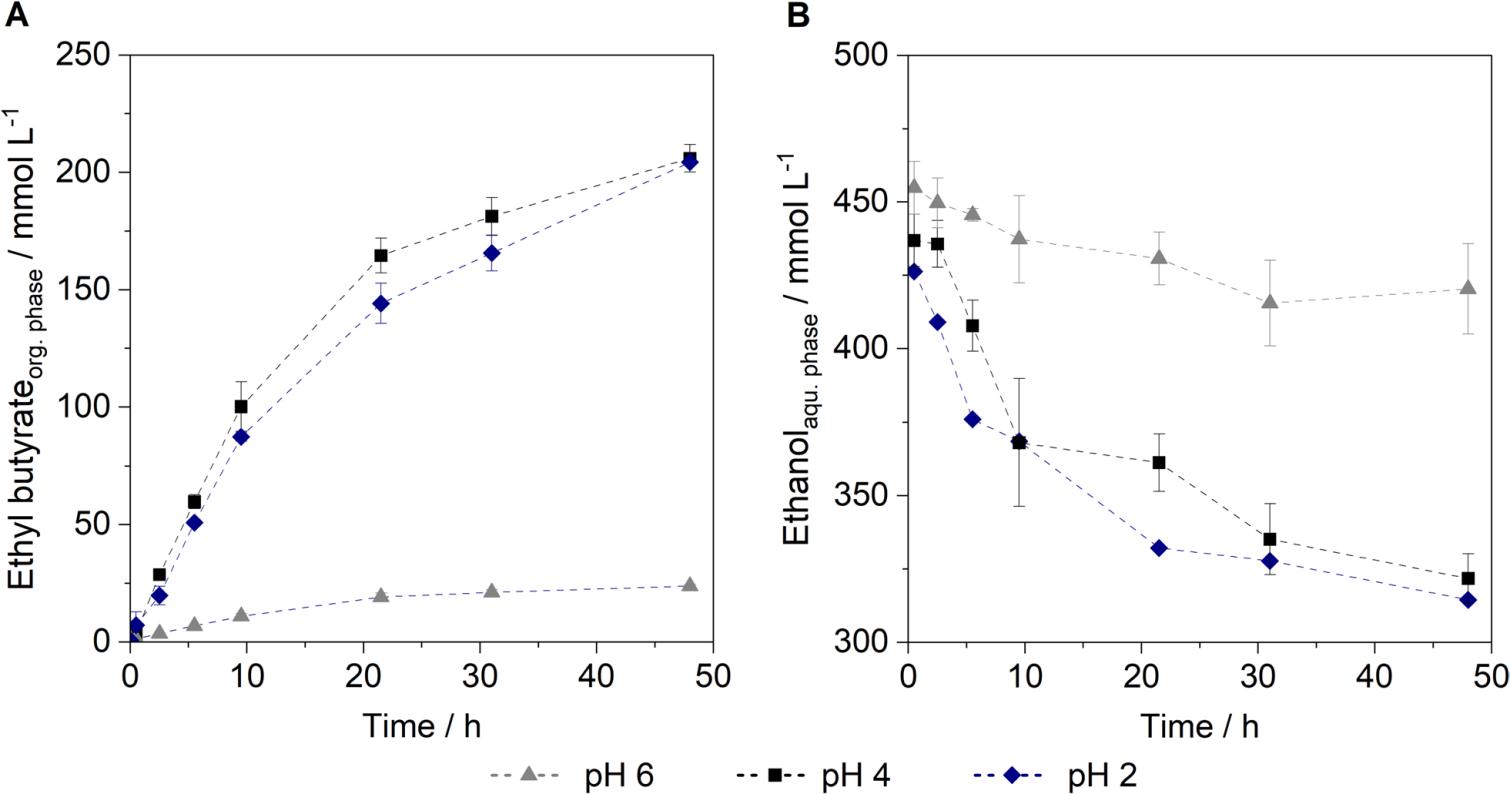
Esterification of ethanol and butyric acid from fermentation broth at different pH. Reaction conditions: 150 rpm, 37 °C, V_aqu._ = 20 mL, V_org._ = 10 mL, organic phase: hexadecane, CALB Novozyme 435 0.5 g L_aqu._^*–*1^, n_biol._ = 3

As an organic phase, hexadecane was added to the unprocessed fermentation broth at a volumetric ratio of 2:1 (aqueous:organic). Hexadecane is immiscible with the aqueous phase and is therefore non-toxic to microorganisms in a two-phase system. The partitioning coefficients of ethyl butyrate, butyric acid, and ethanol were determined as 25.34 ± 5.81, 0.12 ± 0.05, and 0.07 ± 0.05, respectively, in hexadecane. The reaction conditions were adapted to the cultivation conditions to evaluate the potential for simultaneous cultivation and esterification. Simultaneous esterification could enable continuous fermentation by preventing product accumulation, thus making the continuous synthesis of ester more feasible. Esterification experiments were performed using fermentation broth of the fed-batch cultivation at 37 °C and 150 rpm (Fig. 7).

Using a pH of 6 results in a maximum ester concentration of 23.87 ± 0.70 mmol L^–1^. Several authors describe that only the undissociated butyric acid participates in the esterification reaction [10], van den [53, 61]. At pH 6 butyric acid is dissociated to a high extent (pK_a,butyric acid_ = 4.82), indicating that a lower pH is favourable for esterification. Lowering the pH to 4 resulted in a maximum ester concentration of 206.01 ± 5.83 mmol L^–1^ in the organic phase, which corresponds to a mass concentration of 23.93 ± 0.68 g L^–1^. No improvement was observed upon further reduction of the pH to 2. At a phase ratio of 2:1 (aqueous to organic), the ester concentration measured in the organic phase corresponds to twice the substrate consumption in the aqueous phase (Fig. 7B). Ethanol is the limiting component in the reaction. Based on this, a conversion yield of 25.31 ± 0.14% can be calculated.

## Discussion

The numerous applications of bio-based ethyl butyrate increase the interest in an efficient biological production method for this ester [56]. Using a co-culture of *C. tyrobutyricum* and *S. cerevisiae* aims to utilize the complementary metabolic pathways of both microorganisms to enable the one-pot production of ethanol and butyric acid, which serve as building blocks for the enzymatic synthesis of ethyl butyrate.

The production of butyric acid with *C. tyrobutyricum* at a pH between 6 and 8 is an established method [50]. A reduction of the pH to 5, to favour the cultivation conditions of yeast, leads to a significant decrease in butyric acid production (Table 1). Zhu et al. found comparable results at pH 5 using xylose as a substrate [63]. At a low external pH, undissociated acids such as butyric acid and acetic acid diffuse into the cell, where they dissociate [14]. While clostridia are unable to maintain a constant intracellular pH, they intend to preserve a constant transmembrane pH gradient. As a result, the accumulation of H⁺ inside the cell disrupts the proton gradient and the associated oxidative phosphorylation, leading to inhibited cell growth [14]. In addition, Zhu and Yang observed that enzymes involved in the production of lactic and acetic acids exhibited increased activity at lower pH levels, while the enzymatic activity of phosphotransbutyrylase, a key enzyme in butyric acid formation, was reduced [63]. It is therefore essential to control the pH at 6 to ensure a high level of butyric acid production. The ethanol tolerance of *C. tyrobutyricum* is an important factor in the context of co-culture with *S. cerevisiae*. Ethanol is known to cause cell damage by affecting the phospholipid bilayer of the cell membrane, which increases the membrane fluidity [21]. This leads to leakage of cofactors and loss of membrane potential, which is the reason for reduced cell growth [25]. Since the growth of *C. tyrobutyricum* is associated with acid production, it consequently leads to a decrease in acid concentration [50]. However, *C. tyrobutyricum* shows a promising ethanol tolerance as butyric acid production can still be expected at ethanol concentrations of up to 40 g L^–1^. Comparable results were observed with *Clostridium botulinum* at similar ethanol concentrations [42].

In order to establish a co-culture, compromised cultivation conditions must be found. Adapting the cultivation conditions of *S. cerevisiae* to match those of *C. tyrobutyricum*, particularly in terms of temperature and pH, results in decreased ethanol concentrations and yields (Fig. 2). The increased temperature affects the activity of key enzymes involved in glycolysis and results in reduced metabolic efficiency [43]. However, there is no statistically significant difference (paired *t*-test, *t* = 2.04, *p* = 0.07) in ethanol concentration between 32 °C and 37 °C, suggesting that temperature variation did not have a substantial impact under these conditions (Fig. 2C). Compared to the optimal conditions, a significant difference (paired *t*-test, *t* = 2.48, *p* = 0.04) in ethanol concentration was observed due to the additional regulation of the pH to 6 (Fig. 2D). Due to its acidophilic character, *S. cerevisiae* shows better growth and metabolic function under acidic pH conditions [7]. According to Lin et al., a higher pH promotes the formation of organic acids like butyric and acetic acid, which in turn reduces the ethanol yield [30]. Considering the critical role of pH in butyric acid production with clostridia, it will be necessary to regulate pH to 6 during co-culture, even though these conditions resulted in a reduced ethanol yield in the cultivation of *S. cerevisiae*. The production of butyric acid by *C. tyrobutyricum* in the co-culture has an additional negative impact on the ethanol production of the yeast (Fig. 3A). The inhibitory effect of acid is usually explained with the undissociated part of the acid which is able to pass through the membrane by passive diffusion. The acid dissociates inside the cytoplasm resulting in a drop in the intracellular pH that influences the catalytic activity of enzymes and thus inhibits the metabolism [27]. Protons must be pumped out of the cell by the ATP-dependent ATPase, which leads to an increase in the demand for ATP for cell maintenance and a reduction in the supply for biomass formation [24]. This indicates that the inhibitory effect of the acid is pH dependent. Measurements of cell growth under butyric acid stress at different pH levels confirm this assumption, as stronger inhibition was observed at lower pH due to a higher proportion of undissociated acid (Fig. 3B). Maintaining a pH of 6 in the co-culture could therefore positively impact the acid tolerance of *S. cerevisiae*, as, according to the Henderson–Hasselbalch equation, only 6% of butyric acid is present in its undissociated form. Based on these results, cultivation conditions in co-culture were set to pH 6, 37 °C, and 150 rpm.

Considering the different tolerances of the microorganisms to the fermentation products, different inoculation variants in co-culture were tested. Among the inoculation variants, the time-delayed inoculation with *C. tyrobutyricum* proved to be the most promising, with the potential for further increases in product concentrations in fed-batch processes. The concentrations observed here are comparable to those reached in the monocultures with half the sugar concentration (Table 2). These findings indicate that the strains are compatible, allowing for concurrent metabolite production in this inoculation variant. In contrast to separate monocultures, co-cultivation eliminates an additional reaction vessel and additional processing steps such as intermediate product isolation, purification, and mixing, since both precursors are present in a single reactor. *C. tyrobutyricum* benefited from anaerobic conditions established by *S. cerevisiae*, leading to immediate butyric acid production without a lag phase. Additionally, this inoculation variant has the advantage that it eliminates the need for gassing with nitrogen due to the respiratory pathway of *S. cerevisiae*. Glycerol production was observed in all co-culture inoculation variants, whereas no glycerol formation was detected in the monocultures at lower sugar concentrations. The formation of glycerol is expected to result from a stress response of *S. cerevisiae* due to high glucose concentrations at the beginning of the cultivation. In response to the osmotic stress, the yeast increases its glycerol production rate and glycerol is accumulated in the cell [20]. Glycerol consumption was detected in connection with the production of butyric acid (Fig. 4). Oh et al. demonstrated that *C. tyrobutyricum* can consume glycerol as a co-substrate to produce butyric acid, but this occurs only in the presence of acetic acid [40].

A fed-batch strategy was employed to reduce glycerol production and increase product concentration (Fig. 5). Both strains benefit from the fed-batch approach. For *S. cerevisiae*, a decreased glycerol production was found, which contributes to a higher ethanol yield. In the cultivation of *C. tyrobutyricum*, various authors have reported higher yields in fed-batch fermentations than in batch fermentations [18, 50]. The advantages of the fed-batch process were found to include reduced substrate inhibition, lower by-product formation, and increased cell growth [18, 50]. The fed-batch process resulted in a cell density that was more than double compared to the batch process (Figs. 4, A and 5A). The absence of ethanol production after 48 h suggests that the cell count is mainly driven by the growth of *C. tyrobutyricum*. Since *C. tyrobutyricum* follows a mixed-growth pattern, with butyric acid production primarily driven by cell growth, the enhanced cell growth in the fed-batch process leads to higher butyric acid yields [50]. The time-delayed inoculation with *C. tyrobutyricum* proves to be an effective cultivation method in co-culture. However, a disadvantage is the low tolerance of yeast to butyric acid, which limits ethanol production in co-culture. Adapting the cultivation conditions to favour optimal conditions for yeast during the initial 24 h could still improve ethanol production.

The unprocessed fermentation broth from the fed-batch experiment was used to investigate the esterification of the fermentation products. For the esterification step, CALB and an organic phase were added to the fermentation broth to establish a two-phase system. This setup enables the entire process to be performed in a single vessel, thereby reducing infrastructure requirements and potentially lowering overall costs. Ester formation was observed only after a pH shift to 4 was introduced (Fig. 7A). CALB is active over a wide pH range, with its optimal activity typically observed at neutral pH [1]. However, this is usually determined based on its hydrolytic activity. In contrast, the synthesis reaction appears to favour an acidic pH, where the acid remains predominantly in its undissociated form [4, 12]. Based on Buthe et al., only the protonated form of the acid can serve as a substrate in the esterification reaction [4]. This is because the nucleophilic hydroxyl group of the catalytic serine residue cannot efficiently attack the carbonyl carbon of a deprotonated acid, due to the delocalized negative charge on the carboxylate group. Furthermore, it has been shown that at neutral or alkaline pH, the active site of lipases carries a negative charge, which repels the dissociated acid anions and thus prevents their effective binding [39]. Accordingly, the pH of the aqueous phase must be maintained below the pK_a_ of butyric acid of 4.82. Since 87% of the acid is already protonated at pH 4, reducing the pH further does not increase the conversion. As esterification requires a low pH, it does not appear feasible to carry out the process simultaneously during cultivation, given that the conditions differ significantly.

Despite this shift in pH, the conversion yield remains limited to around 25% (Fig. 7B). Lu et al. reported a comparable yield of 32.8% in a two-phase system with dodecane (organic:aqueous 1:2) for ethyl butyrate synthesis using CALB [33]. The formation of longer-chain esters like butyl butyrate appears to be thermodynamically more favourable, leading to yields approximately twice as high [33]. Several factors may contribute to the incomplete esterification. The main limitation of the system is the high water content, which shifts the reaction equilibrium to favour the hydrolysis reaction. To maximize the conversion, the reaction is usually carried out in an organic solvent with minimal water content, often combined with water removal strategies [19, 32]. However, the esterification of ethanol and butyric acid in the untreated fermentation broth is possible with the addition of an organic solvent. As the other fermentation products remain in the aqueous phase due to their partition coefficient, this approach even allows in situ product recovery of the ester. While this study focuses on the production of ethyl butyrate, comparable studies primarily explore the production of butyl butyrate. Table 3 summarizes comparable systems that rely solely on microbial product formation without any substrate supplementation for esterification.

**Table 3:** Overview of comparable microbial ester production systems reported in literature

Biocatalyst	Max. product conc./g L¯^1^	Lipase	Extractant (aqu.:org.)	Ester_aqu. phase_/g L¯^1^ (mol L^−1^)	Ester yield_P/S_/mol mol^−1^	References
*S. cerevisiae*,* C. tyrobutyricum*	18.6 ethanol, 45.6 butyrate	*Candida antarctica* (Novozyme 435), 2.5 U mL^−1^	Hexadecane 2:1	Ethyl butyrate 12.0 (0.10)	0.14	This study
Engineered* C. tyrobutyricum*	6.0 butanol, 16.3 butyrate	De novo synthesis in *C. tyrobutyricum*	Hexadecane 10:1	Butyl butyrate 14.26 (0.10)	0.28	Guo et al. [23]
2 engineered *E. coli* strains	4.7 butanol, 5.2 butyrate	Candida sp. (Lipozyme CALB L), 25 U mL^−1^	Hexadecane 1:1	Butyl butyrate 7.2 (0.02)	0.15	Sinumvayo et al. [48]
*C. tyrobutyricum*,* C. acetobutylicum*	Not specified	Surface-displayed lipase	Dodecane 2:1	Butyl butyrate 6.7 (0.04)	0.14*	Lu et al. [33]
*C. tyrobutyricum*,* C. beijerinckii*	6.9 butanol, 9.7 butyrate	Candida sp. (Lipozyme CALB L), 25 U mL^−1^	Hexadecane 2:1	Butyl butyrate 5.1 (0.03)	0.11*	Cui et al. [10]
*C. acetobutylicum, Actinobacillus succinogenes*	4.8 butanol, 4.8 acetate	*Candida antarctica* (Novozyme 435), 100 U mL^−1^	Dodecane 2:1	Butyl acetate 2.2 (0.02)	0.05*	Lv et al. [35]

^*^calculated from given data; aqu.: aqueous phase, org.: organic phase

The co-culture system developed in this study achieves the highest product concentrations of alcohol and acid compared to other reported biocatalyst combinations, highlighting its superior potential for short-chain ester synthesis. The use of a co-culture presents clear advantages over a single engineered strain, as it allows for the selective optimization of both target products. To compare ester concentrations at different organic-to-aqueous phase ratios, ester concentrations were calculated based on aqueous phase volume. The ester concentration of 11.96 g L⁻^1^ achieved in this study exceeds those reported in similar studies where acid and alcohol were obtained through co-cultivation [10, 33, 35, 48]. Additionally, this study required a significantly lower enzyme concentration. However, Guo et al. achieved slightly higher butyl butyrate titers through de novo synthesis in *C. tyrobutyricum* [23]. The ester yield aligns with existing co-culture systems that produce butyl butyrate. However, there remains considerable potential to improve the yield by increasing the conversion rate, as a significant amount of substrate remains unutilized. Additionally, in a very recently published study, ethyl butyrate production from corn stover was investigated for the first time, and a maximum yield of 30.55 g kg¯^1^ was achieved [57]. The microorganisms employed in this study also demonstrate potential for growth on alternative substrates which could be explored in future research to enhance process sustainability and reduce production costs.

## Conclusion

The co-culture of *C. tyrobutyricum* and *S. cerevisiae* successfully enables the one-pot production of butyric acid and ethanol yielding final concentrations of 45.62 ± 3.82 g L¯^1^ and 18.61 ± 4.11 g L¯^1^. The results highlight the importance of optimizing inoculation timing and feeding strategies to balance the production of both target products while mitigating the inhibitory effects of each microorganism on the other. Delayed inoculation with *C. tyrobutyricum* facilitated ethanol production despite the yeast’s low acid tolerance. The implementation of a fed-batch method led to an increase in product yield and minimized by-product formation. Esterification of the fermentation products in untreated fermentation broth, coupled with in situ extraction of the ester, was successfully achieved by adding an organic phase and reducing the pH to 4. As a result, a maximum ester concentration of 23.93 ± 0.68 g L¯^1^ can be attained. Optimizing the esterification process will be a focus of future work. Overall, this co-cultivation strategy represents a promising approach for the synthesis and recovery of bio-based ethyl butyrate.

## Data Availability

No datasets were generated or analysed during the current study.
